# Essais sur la Méthode Sous-Cutanée

**Published:** 1842-01-01

**Authors:** 


I. Essais sur la Méthode Sous-cutanée. Par le Docteur *Jules Guerin.* Paris, 1841, pp. 126.

Essays upon the Subcutaneous Mode of Operating.

II. Memoire sur l'Etiologie Generale des Deviations Laterales de l'Epine. Par le Docteur *Jules Guerin,* 1840.

Essay upon the Etiology of Lateral Curvature of the Spine.

III. Recherches sur les Luxations Congénitales. Par le Docteur *Jules Guerin.* Paris 1841.

Researches upon Congenital Dislocations.

IV. Memoire sur l'Intervention de la Pression Atmospherique dans le Mecanisme des Exhalations Sereuses. Par le Docteur *Jules Guerin.* 1840.

A Memoir upon the Influence of Atmospheric Pressure in the Production of the Serous Exhalations.

V. Memoire sur un Cas de Luxation Traumatiqe de la Seconde Vertebræ Cervicale, datant de Sept Mois. Par le Docteur *Jules Guerin.* Paris, 1840.

Relation of a Case of Dislocation of the Second Cervical Vertebra of seven months' duration, successfully treated.

These pamphlets have appeared during the last two years in the pages of the French Medical Gazette, and have been noticed from time to time in the Periscope of this Journal;* but their republication in a separate form, and the importance of the subjects to which they relate, demand at our hands a rather more detailed view of their contents than we have hitherto given. Three of the memoirs form part of a dissertation upon the defor-

---

* See Nos. 62, 64, 65, 66, 67.

mities of the osseous structure, which M. Guerin is now republishing, and to a portion of which we referred in our number for July last. The author is a bold and able surgeon, as apt with the pen in recording as with the bistoury in performing his various operations, and, certainly, if the results he declares he has obtained be confirmed by the experience of other practitioners, much of our surgical practice is destined to undergo considerable modification and extension.

### I. THE SUBCUTANEOUS MODE OF OPERATING.

"It is," says Mr. Lawrence in his Lectures, "the boast of modern surgery to have diminished the number of operations. I speak within limits when I assert that there are not so many operations performed now, by one half or two thirds, as when I first began to study the profession. This important difference to which I allude has arisen from the improved knowledge of the nature and treatment of disease." It would be well if some of those who have been seized with the prevalent mania for operating, would bear this observation in mind. It is indeed unquestionable, that many deformities have of late been discovered to depend upon perverted muscular action, and to be capable of relief by the division of the muscles or their tendons implicated, and that such division is not attended with the ill-consequences heretofore believed to result from meddling with these structures. But, when we observe the number of cases of clubfoot, strabismus, stammering, distortions of the spine, &c. which have latterly been submitted to the knife, we are tempted to believe that too little discrimination has been employed in the selection of cases likely to be benefitted, and, that a great deal of enthusiasm, and some charlatanism may have obscured the due consideration of the principles upon which operations should alone be undertaken. Upon what principles surgeons are enabled to boast their two or three hundred cures in the brief period of a year or two, we are at a loss to comprehend, at least if permanency is to be considered as one of the elements of a cure. If our predecessors in surgery performed more of the acknowledged operations than our improved knowledge allows us to undertake, they were at least far more careful in their institution of new ones; and before they recommended these for general adoption, examined all the circumstances connected with them with scrupulous care and exemplary patience. There can be no doubt, as we have already observed, that many of the deformities alluded to are relievable by the operations now in vogue; but, there can be as little doubt, that many which have been submitted to them never should have been so; and, that a most unwarrantable precipitancy in undertaking new cases, prior to a patient observance of the results of the first ones, has been committed.

However, if M. Guerin's observations upon "Subcutaneous Surgery" be correct, the multiplying of these operations is comparatively of no consequence, as the most formidable of them may be undertaken without any danger, and almost without inconvenience. He prefaces this republication of his views with an introductory chapter, in which he establishes his claims of being the first to prove that wounds, to which the access of the air is prevented, healed at once without any inflammatory or suppurative process. It is true the harmlessness with which the subcutaneous section of the tendo-Achilles may be performed has been long known, but, even the most recent operators, as Dupuytren and Stromeyer, merely practised this as an empirical operation, believing that, by avoiding exposure, the suppuration or exfoliation of the tendon was prevented, but never referring this to any general theory or explanation. So, too, Dieffenbach attributed the success to the mildness of the inflammatory action, and never for an instant supposed that no inflammation whatever was present. M. Guerin first divided tendons in 1836, but did not permit the bistoury to pierce the skin a second time, viz. at the point where the incision terminated. Finding the one puncture sufficient he was struck with the ease with which the wounds united, and the little trouble they caused; but, so little did he himself at that time suspect the principle involved, that he, from dread of the operation, deferred for two years dividing the spinal muscles for those deformities dependent upon their contraction. The division of tendons by some other surgeons was not attended by the immediate union which followed his own operations, and serious consequences sometimes resulted. Upon reflection he discovered that these ill effects arose from the apertures of the skin being too large, and too directly over the incision of the tendon, and that the instruments employed were far too broad. Experiments upon animals, and subsequent experience, led him to draw the conclusion, that the absence of ill effects after division of tendinous structures, did not arise, as supposed, from the slight re-active power of the wounds of tendon, but from the exclusion of the air—an exclusion that produces what he terms "immediate organization," which is not only distinguished from, but impeded by the presence of inflammatory action.

"What do my experiments upon animals and my operations upon man shew? The former, made and repeated many times, comprehending subcutaneous incisions of all dimensions, from 1 to 30 centimetres,* practised upon the limbs or trunk, single or multiplied, involving by turn tendon, muscles, nerve, vessels of small calibre, and even the bones themselves: these attended sometimes with effusions of blood, and performed under every possible combination, exhibited, at all times, an utter absence of suppurative inflammation, and the presence of 'immediate organization.' Sometimes, the incisions extended from the nape of the neck to the sacrum, traversing the muscles, vessels and nerves of the spine, the animal remaining as calm as if suffering from a mere scratch. Sometimes the wounds were far deeper and more severe, but never were they followed by suppuration............. Is there a surgeon who would have dared, at the same sitting, to have performed upon one individual the subcutaneous section of more than forty muscles and tendons? That have I dared to do without hesitation or bravado, resting upon the truth of the principle laid down; and do not the boldness and novelty of my attempt, and the surprise and incredulity it excited, present us with some idea of the originality and certainty of this principle?...... I may demand of the incredulous, to cite one solitary fact of suppuration ever having followed any of my subcutaneous operations; yet have these now amounted to more than *two thousand*, and I have performed publicly more than 500 since my election to the hospital for children."

He reasonably objects to the want of success of those, who do not adhere rigorously to his method, being laid at his door. The work itself

* A centimetre is inch 0.394.

consists of two Essays; the first relating to subcutaneous wounds in general, and the second to those of the joints in particular. An Appendix contains a few cases recently treated by some other surgeons.

I. *On Subcutaneous Wounds.*

A. *Experimental Part.*—In this the author relates the various experiments he made upon dogs prior to trying the operations upon the human subject, and the results of these latter. A detail is unnecessary; suffice it to say, some of the most important muscles of the body were among those divided, and that the incisions frequently extended from 8 to 10 centimetres in length, and from 4 to 5, or on some occasions even to 10 or 12, centimetres in depth. A considerable effusion of blood beneath the skin and between the lips of the wound at once occurred, but a small piece of adhesive plaster, and the employment of slight pressure, were the only means had recourse to. No local inflammation or general fever ever resulted. The patients after the third day were enabled to arise, and when supported to walk, and to employ the various mechanical means necessary to complete the removal of the deformity.

B. *The Theory of the Immediate Organization of Subcutaneous Wounds.* What theory the author may have in his mind we know not; but, all he expresses is the simple fact, that the exclusion of the air permits, and its admission prevents wounds undergoing the process of *immediate organization,* as he terms it, though, in what this mode of healing differs from that of union by the first intention, we are at a loss to perceive.

C. *General Consequences and Applications.*—M. Guerin observes, that all wounds, however they may differ in their early stages, according as they present the adhesive or suppurative forms of inflammation, resemble each other in their latter stage—that of cicatrization. Cicatrization takes place speedily in proportion as the air is excluded, and even in suppurating wounds this process only occurs after such exclusion; in these this is effected by means of the membrane, first described by Bichat as existing upon suppurating wounds, and afterwards denominated the puogenic membrane—the formation of pus not being, however, a special secretion, requiring any special organ, but a mere modification of the blood, transuding through this membrane into contact with the air.

The practical applications of the doctrines brought forward in this essay are indeed important. In the treatment of wounds, the importance of bringing all the parts into accurate contact, and of effectually closing their orifices, is at once seen. Wounds of the great cavities of the body, for example, should always be treated upon the principle of obtaining immediate union. Experience alone will demonstrate all the various cases in which the subcutaneous mode of operation may be employed, but many may even now be indicated. Thus, a vast number of operations upon tendons are performed with success, which formerly never could have been attempted; congestive abscesses forming in the groin, thigh, or lumbar region, have been opened in several individuals suffering from tubercular affections; the sphincter has been thus successfully divided for fissure of the anus; various serous and sanguineous subcutaneous effusions have been dispersed, and an exostosis has been removed from the tibia. In fact the original expectations of M. Guerin, as contained in the following passage, seem to be in progress of realization.

"We may expect that the liberation of certain inflammatory engorgements, the division or removal of certain tumors, the opening of some descriptions of cysts and abscesses, will, through the subcutaneous mode, be effected without consecutive inflammatory accidents. Lastly, as the most important application, I will allude to the subcutaneous liberation of crural and inguinal herniæ, and their radical cure by the adhesive occlusion of their orifices."

Other surgeons have now borne testimony to the efficacy and safety of subcutaneous surgery in some novel applications. MM. Barthélemy and Malgaigne have employed it with success in the incision of synovial cysts developed around the joints. Lisfranc and Pinel Grandchamp have removed considerable ganglia; M. Lisfranc met with neither success or accidents, M. Pinel Grandchamp with a marked success. M. Ricord has successfully applied the subcutaneous method to the treatment of a varicocele by ligature of the veins. M. Dufresse-Chassaigne has treated a case of loose cartilages of the knee. M. Jobert has attempted the radical cure of hydrocele by means of subcutaneous incision of the tunica vaginalis.

### 2. *Subcutaneous Wounds of the Articulations.*

This essay detailing the application of the above doctrines to operations about the joints, need not occupy us long, it having been already noticed in this Journal.* It is sufficient to say, that, guided by some successful experiments upon dogs, M. Guerin eventually obtained the following results.

"It will be seen from a memoir which I addressed to the Academy during its last sitting, that I have already frequently performed the subcutaneous section of the ligaments of the knee and ankle joints, to remedy various deformities arising from their undue contraction. In many of these operations, and especially in the division of the tibio, and perineo-tarsal, and the tarsal ligaments, I have been obliged to extend my incisions within the interior of the articular cavities. These operations, repeated many times, have never given rise to any inflammatory accident."

By the subcutaneous method, serous, sanguineous, or purulent collections may be evacuated from the articulations; and the author anticipates that, when our diagnosis of the exact state of the joints in anchyloses is more exact, many of these cases will be found amenable to the same proceedings. Another application has even been sometimes successfully put into force, viz. the section of the ligaments and capsules in irreducible and congenital dislocations.

"In fact, I have been enabled by this method to cure a congenital dislocation of the sternal extremity of the clavicle in a girl 11 years of age. This deformity had been submitted in vain to all kinds of treatment. The projection of the sternal extremity of the clavicle was very considerable, and the ligamentous connexions were in a very relaxed state. I made, under the skin, incisions around the articulation, so as to include the ligaments and capsule, and circumscribed by numerous and deep scarifications the field of movement of the inner extremity

* No. 66, p. 519, Oct. 1840.

of the clavicle. After keeping the joint for ten days perfectly at rest, I found the extremity of the clavicle imprisoned, as it were, within a rim of recent formation, having also contracted adhesions with surrounding parts, calculated to prevent a recurrence of the displacement. I thought it would make matters still more certain if I repeated the operation. After a month's rest and precautions, the girl was enabled to execute with her arm the various movements, which had heretofore always been accompanied by a considerable projection forwards of the extremity of the clavicle."

### II. THE ETIOLOGY OF LATERAL DISTORTION OF THE SPINE.

" This Essay contains an application, to the study of lateral distortion of the spine, of the doctrines I have already delivered respecting the etiology of club-foot.* In this point of view, the curvature of the vertebral column may be considered as the club-foot of the back, that is to say, the contracted state of the muscles (retraction) produce in the spine, as in the foot, anormal changes of direction and form, modified indeed by the particular condition of the parts in question, and by the different directions in which this contraction takes effect. Thus, it is merely the same fact, which we have broken up into sections, but whose various parts will be found to unite and blend themselves into a perfect unity. Club-foot, spinal curvature, wryneck, congenital dislocations, and many monstrosities, are but the partial and varied effects of the operation of one cause, just as the different varieties of congenital club-foot, equinus, varus, and valgus, offer on a less scale evidence of a single and similar cause operating differently upon the muscles of the leg and foot."

After alluding to a former communication to the Academy, in which he detailed the successful result of his sections of the spinal muscles in lateral distortion, the author applies himself in the present memoir to the demonstration—that the muscles of the spine, influenced by certain states of the nervous centres, take on involuntary contraction, and, remaining permanently shortened, by the traction they exert upon the portions of the osseous system to which they are attached, produce the deformity in question. He proposes certain queries, the answers to which form the illustration of his subject.

1st QUESTION.—*Do there exist examples of spinal distortion, accompanied by, and evidently caused by sensible alterations in the condition of the nervous centres?*

The answer is in the affirmative; for (1st), in the fœtal state, and especially in the case of monsters, distortions of the spine, accompanied by various deformities of the joints, are found to co-exist with more or less extensive changes in the nervous system. In the author's work on deformities of the bones, to which the prize of the Academy was adjudged, he has collected a series of evident changes in the brain and spinal-marrow, from their complete destruction down to the minutest anormal appearance. Deformities of the skeleton were found to exist in proportion to the gravity of these changes of the nervous centres; thus, partial or entire loss of the brain, or spinal-marrow, were accompanied by the most complete distortions of the limbs and excessive curvature of the spine. These defor-

* Medico-Chirurgical Review, No. LXV., p. 226.

mities are not only co-existent with, but are subordinate to the lesions of the nervous system, as may be seen by examining the manner in which they are produced; for, the muscles are found to be shortened, hard, and tense in the direction of the various deformities, forming the cords to the bowing of the different angles and curvatures. Anormal flexions of the limbs, luxations, and deformities of the joints, then, are found in the fœtal state (in animals as well as man), and always in due correspondence with the extent of the nervous lesions.

But (2) these observations need not be confined to the fœtal state; for the production of spinal deviations by muscular contraction, originating in nervous affection, is capable of ample demonstration in after-life. M. Guerin has observed at all periods, from the first few days after birth to adult age, curvature of the spine following cerebral or cerebro-spinal affections, and accompanied as in the fœtus by numerous deformities of the articulations: while, in proportion as the cerebral affection has been slight, so has been the accompanying deformity. These facts have been observed too frequently in young children to be other than rigorously exact, and the same observation might be extended through all the years of childhood to adult age. He possesses many cases of the deviation of the spine having supervened in girls 15 or 16 years of age, after various convulsive affections of the brain and spinal-marrow.

2d Question.—*In the absence of sensible alterations in the nervous centres, are there any certain means of recognizing that a curvature of the spine is produced by active muscular retraction, originating in an affection of the nervous system?*

This question may be also answered in the affirmative. The positive proof in these cases is of course not always to be obtained, as the majority of them are yet living; but the question is whether material exists to enable us to form our diagnosis independently of an autopsy: this may be considered in two points of view.

1. The spinal distortion may be accompanied by other symptoms of the nervous affection, which has given origin to it; and indeed it is rare for the nervous affection to confine its manifestation to the exciting muscular or spasmodic contraction, or for such contraction to be limited to the spinal muscles. And, thus, besides the changes in the physiognomy and gait of persons suffering under cerebral or spinal disease, we usually find undue contraction of other muscles, as wryneck, distortion of the feet or hands, permanent flexion of the extremities, &c. In such cases we should not detach the spinal deformity and explain its production by a separate agency, but consider it as a part of the *tout ensemble* of the signs of the affections of the nervous centres. M. Guerin says that such cases are constantly occurring to him, but, that it is in the hospitals devoted to affections of the nervous system, as the Bicètre and Salpétrière, that we see hemiplegia, paralysis, and every form of nervous affection, accompanied by every variety and every combination of muscular contraction, producing deviation of the spine among other distortions.

2. But in other cases no such coincident manifestations exist, and such cases present some difficulty: here the nervous affection is confined in its influence to one or two points of the circumference, or may exist in, and be circumscribed within the nerves themselves, which supply the spinal muscles. Affections of the spine may, in fact, occur after very local and slight affections of the nervous system, but in determining that they do so arise, M. Guerin seems to us completely to fail, he being obliged to throw himself upon remote analogies, or, in other words, merely describes the actual condition of the parts. He says that all the characters of these spinal curvatures resemble those found in cases avowedly produced by nervous lesion, and that this identity of effect must arise from an identity of cause. He lays great stress upon the fact, that the muscles are in a state of active retraction, very different from that of mere passive or consecutive retraction; the one exerting a powerful force upon the points of attachment, and approximating them in various directions; the other being merely secondary to such approximation, they possess no tension, and merely fill without limiting the space: in the former, by reason of the resistance they meet with, they become of a fibrous consistence, while in the latter they degenerate into a fatty condition: in the one they are firm, bundled well together, and resisting under the skin—sometimes being as hard as fibro-cartilage; in the other, they present slight resistance and an anormal softness. The active retraction may be limited to a single muscle, or even to a portion of the same, and thus it and paralysis with atrophy may occupy different portions of the same muscle. Thus portions of the trapezius have been found contracted and fibrous by the side of other paralysed, wasted, and membranous portions, and both in connexion with muscles in their normal condition. So too the longissimus dorsi has been seen actively retracted, while the sacro-lumbalis by its side has been so merely passively. These varieties of texture and form of the parts often throw great light upon the nature of the case. The appearances now alluded to are however not always very evident, and may be masked by others.

3d QUESTION.—*What are the different varieties of this active muscular retraction, and within what limits are we to consider it as a cause or active element in the production of these deformities?*

Persons who have paid much attention to the effect the nervous affections, which manifest themselves by convulsive movements, exert upon the development of the organization, will be in a condition to answer this. It is well known that, after the various diseases of infancy, in which a convulsive affection has been even slightly evident, a mere passing event, how frequently some muscle of the neck or limbs remains contracted for a shorter or longer period. Such effects, which are observed in muscles whose action is so visible as those of the neck or arms, are also to be found existing in the spinal muscles, as indeed might have been inferred, from the great number and intimate connexion with the spinal-marrow of the nerves which supply them. If we do not observe the spinal distortions develope themselves as soon as the wryneck, flexion of the elbow, or clubfoot, it is not that they depend upon a different cause, but that the spine offers a greater resistance, and that its muscles, more closely confined in their sheaths, do not project so prominently. Here, as in clubfoot, the retracted muscles become struck with a species of paralysis, which prevents their continuing to acquire development in proportion to the parts of the skeleton to which they are attached. They remain proportionally shorter, and distortion is the necessary result.

But to these cases, which may be called the results of an internal malady, compromising the brain or spinal-marrow, are to be added others which result from external causes, applied directly to the muscles themselves, or rather to the nervous elements which animate them. It has long been well known that wounds, sores, or abscesses of the calf produce a shortening of the muscles which compose it, and that this shortening gives rise to a permanent extension of the foot upon the leg. So, too, persons accustomed to treat affections of the spine are well aware, that deviations of the column often follow wounds or blows of its muscles. In these cases (and where curvature results from various diseases, as rheumatism, &c.), although the muscular lesion is the only one evident, yet we are justified in referring the contraction of the muscle to an affection of the nerve which supplies it; for it is from the nerves alone the muscles derive their motility, and the best understood nervous maladies modify this motility, from convulsive contraction to actual paralysis. Even when the distortion may arise from other accidental causes, muscular contraction much influences its eventual progress.

4th Question.—*Are there any certain means of distinguishing distortion produced by active muscular retraction, from distortion resulting from other causes?*

M. Guerin states that, in his work upon deformities, he has proved that there may be passive muscular distortion of the spine, active muscular distortion, (the most frequent variety,) and osseous distortion. Each of these causes manifests itself by the peculiar nature of the effects it produces, by attention to which the diagnosis may be pronounced. But distortions of the spine, however originating, present certain phenomena in common, arising from the endeavours of the muscular system to re-establish the equilibrium of the body, when in the erect posture. General muscular debility, an imperfect antagonism of corresponding muscles, paralysis of certain of the muscles, a primary inequality of the two halves of the skeleton, rickets or scrofula, any of these causes may operate in determining the spine from its vertical position. All these causes are attended, like active muscular retraction, with their peculiar signs. Thus, muscular and ligamentous debility impresses its marks upon all the muscles, ligaments, and articulations, as it does upon the spinal column; and the distortions arising from rickets or scrofula are but some among the various local visible effects of these diseases. For a detail of the specific characters of each variety the author refers to his former work. But there are certain characters produced by degrees of active muscular retraction which are common to all cases of distortion, although the degree in which this retraction mingles with the various other signs is not always easy to determine. When once, by the operation of any of the causes to which allusion has been made, the spine commences departing from the perpendicular, a species of struggle ensues between this pathological cause and the action of the muscles of the spine endeavouring to maintain the centre of gravity duly poised, whence result the *alternate* curvatures always to be observed. These muscles exert then a retractive power analogous to primitive muscular retraction, and, although the origin of such retraction is different, its effect in shortening the muscles, and acting upon the skeleton becomes the same. To this form Guerin gives the name of *active secondary* retraction, thus distinguishing it on the one hand from the *primary active* retraction, and, on the other, from the merely *passive* retraction, which adapts the form of the muscles to the spaces they have to occupy.

Such is the theory of our author, justificatory of the numerous operations he has of late performed for the removal of distortions of the spine. Doubtless some cases may be so explained, but numerous objections arise, had we space to allude to them, to its adoption as a general explanation. So, too, do we feel as yet very sceptical as to the incision of the affected muscles proving a *permanent* cure in any considerable number of cases. It will require a few years longer yet to determine the true value of this, and some other operations of recent introduction.

### III. CONGENITAL DISLOCATIONS.

Congenital dislocations form another example of the effects produced by what the author terms a primary active muscular retraction. He divides the memoir into two parts; the one termed the scientific or theoretical portion, comprehending the etiology, topography, and mechanism of the production of these affections; the other, or practical portion, embracing the consideration of all the pathological circumstances, influencing the permanent reduction of the dislocations, and of the means whereby this is to be effected.

### 1. *Theoretical Portion.*

A.—*Congenital Dislocations, like Club-foot, Wryneck, and Spinal Distortion, are produced by active muscular retraction, operating in different modes and degrees.*

The same line of demonstration employed in reference to spinal distortion, is here followed out. In monsters, dislocations are found to exist in common with other effects of perverted muscular action, and in proportion to the extent of lesions of the nervous system. Among the preparations exhibited to the Academy, was one of an acephalous fœtus, in which the head was completely drawn back, the spine much curved, and all the principal joints dislocated. This he considers a truly pathological proceeding, and not owing to (as according to the popular theory) an arrest of development, with *consecutive* retraction of the muscles. The lesions of the brain, found in monsters, do not arise from absence of development, for the parts awanting have existed, and are in a pathological process of removal; the muscular contraction is also very different from that which is merely passive and consecutive, for the muscles are not merely shortened between their points of insertion,—they are so often to a third or fourth of their normal length,—but they are also in such a state of undue tension, that the portions of the osseous system to which they are attached necessarily become distorted or broken. Their texture, too, which has become hard and fibrous, is very different from the fatty degeneration of consecutively shortened muscles.

To shew how little influence pressure, position, or other accidental circumstances have in producing these dislocations, we may observe, that the same luxations often exist, and in the same direction, upon the two opposite sides of the body: that they are frequently found to surpass in extent dislocations produced suddenly, and that their direction is determined by the direction of the retracted muscles, which is often anormal.

B.—*All the Deformities comprised in the category of Congenital Luxations, Sub-luxations, and Pseudo-luxations, are, as the varieties of other articular deformities, produced by active muscular retraction or paralytic contraction, differently distributed, combined, and graduated in the muscles subservient to the articulations.*

Congenital dislocations may be complete or incomplete,—luxations and sub-luxations, or they may be simulated by other deformities,—pseudo-dislocations.

It was an error formerly to attribute these varieties to different causes: they all arise from muscular contraction, but from different distributions, degrees, and combinations of this retraction, the luxation being complete or not, accordingly as the muscular retraction is powerful or affects many muscles. These two degrees of dislocation exist at different periods of fœtal life. Complete dislocation is found at the earliest period, when the articular cavities are insufficiently developed, the adaptation of their surfaces incomplete, and their attachments lax; but, in the progress of fœtal life, as the cavities become deepened, and their surrounding capsules firmer, sub-luxation and pseudo-luxation predominate. Pseudo-luxations are properly not luxations at all, although they bear some outward resemblance to them, and consist of an anormal direction imparted to the bone by muscular retraction, without actually displacing it from the joint.

Congenital luxations may arise *directly* from primary retraction, and spasmodic contraction of the muscles, or they may arise *indirectly* from the paralysis of certain muscles, whereby their antagonists acquire an undue power.

C.—*Congenital Dislocations may occur successively or simultaneously in every joint of the body, from the lower jaw to those of the bones of the feet.*

Under this section the author presents us with a list of the different dislocations he has met with. The length precludes our attempting any analysis, and it will suffice to say there is hardly a joint in which he has not found several varieties of luxation to occur.

M. Guerin does not deny that congenital dislocation may occasionally occur from other causes besides muscular contraction: thus, diseases of the joints, occurring during fœtal life, sometimes terminate in displacements, but the characters of these are quite different from those of the true congenital dislocation. He adduces an example of dislocation of the femur. The surrounding muscles were even scarcely passively shortened, while the articular cavity, in part destroyed, was found filled with a fibro-cartilaginous structure.

D.—*The progress and completion of Congenital Luxations are subjected to the influence of the same accessary causes as Club-foot, Spinal Distortion, &c. viz. an arrest in the development of the retracted Muscles, the physiological contraction of the Muscles, and the vertical action of the weight of the Body.*

It is rare for a luxation to become at once complete, although there exist examples in which energetic contraction of the whole or chief portion of the muscles of the articulation have produced it; this happens usually at the earliest period of intra-uterine life, and implies a very severe lesion of the nervous system. Under what influences then do subluxations become complete ones, and do pseudo-luxations simulate true luxations, which is usually not the case until some period of extra-uterine life? There are three auxiliary or complementary causes of these changes, viz.

1. *The arrest in the development of the muscles.*—This is an early effect of retraction, whereby the disproportion between the muscles, and the portion of the skeleton to which they are affixed increases, and the dislocation, at first partial, becomes complete: this occurs, however, much more easily in some joints than in others. 2. *The physiological or natural contraction of the muscles* continuing to act in the direction in which the original displacement has occurred, contributes to increase it. The points of insertion of the muscles being now brought into different relative positions, their lever power or mechanical effect frequently becomes quite changed. Thus, when the femur is luxated upwards and outwards, the adductors, semi-tendinosus, and semi-membranosus assist in the displacement vertically, in consequence of the absence of a point of resistance to the superior extremity of the femur, and laterally by an increase of their angles of insertion. 3. *The vertical action of the weight of the body* tends incessantly to augment the displacements of the lower extremities. Thus, in the dislocation of the hip, which is usually double, the weight of the body tends to force the pelvis down between the two femoral heads. By reason of these accessary circumstances the progress of the dislocation is gradual but continuous, and, so numerous may be the shades of difference occurring from the operation of various circumstances, that it is impossible to pronounce when a partial dislocation may become a complete one: but a luxation of the femur, which may be cited as an example of the others, is rarely completed before the third or fourth year—a fact which has deceived many surgeons, leading them to believe the luxation was not congenital, but arising from circumstances operating after birth.

E.—*Congenital Luxations present (besides their proper and various mechanical characteristics) specific, general and local characteristics, which are common to them all, and are but a repetition of those of other Congenital Deformities.*

The specific characters are—1, *local,* viz. the retracted state of the muscles, producing a hard prominent, tense bundle, limiting the movements of the parts, and changed more or less into a fibrous consistence: 2, *general,* to which allusion has been made in speaking of spinal distortions, as traces of an affection of the nervous centres, shewn by the physiognomy or the contracted state of other muscles, &c.

F.—*The Therapeutical Indications are derived from the consideration of the Etiology; and especially consist in the subcutaneous section of the retracted Muscles, and in the employment of appropriate mechanical means for the effectual and permanent reduction of these Luxations.*

The section of the muscles of the thigh may be made with the same safety and the same success that have attended the operations upon the spinal muscles and other parts. But, as the incisions are both deep and considerable, the subcutaneous plan must be rigorously followed out, if suppuration is to be avoided, and immediate organization achieved. In nine cases M. Guerin has performed the operation for reduction of these luxations, and with an immediate removal of the principal obstacles to the reduction. Many cases require much subsequent adaptation of various mechanical means, and some are incurable. The consideration of the reductibility of these affections belongs to the second part of the essay.

### 2. *The Practical Portion.*

CHAP. 1.—*The Anatomical Conditions which facilitate, render difficult, or prevent the permanent Reduction of the Dislocation.*

These conditions are to be found—1, in the state of the muscles and other soft parts surrounding the luxated part;—2, in the state of the articular surfaces themselves, and in that of the neighbouring parts of the skeleton;—3, such as the formation of new articular cavities and consecutive deformities of the bones.

### A.—*Changes in the Muscles and other Soft Parts.*

A. *The Muscles.*—These become *shortened* not only by the active retraction which produces the dislocation, but also secondarily and passively by reason of the approximation of their points of insertion. In complete luxation the greater part of the muscles which influence the articulation are primarily contracted; but, in sub-luxation, or pseudo-luxation, the number of muscles influenced by either forms of retraction varies considerably—a solitary bundle may be submitted to primary retraction, and a great number affected secondarily, or *vice versa.* The *direction* of the muscles often becomes changed by the alteration of the position of the osseous levers. The *texture* suffers three modifications: the muscles may become "fibrous," owing to the unnatural and permanent traction to which they are subjected; those of them which have remained in a state of inertia and relaxation, owing to their points of insertion having been preternaturally approached, undergo a fatty degeneration; while, others again, which are brought into increased exertion, to supply the place of those which are thrown by the dislocation into a state of desuetude, become hypertrophied.

The effects of the muscles upon the reductibility are—1. Those which are primarily contracted, or have passed into the fibrous state, constitute insurmountable obstacles to the reduction. Their mechanical extension can give them only for the moment a length sufficient to re-establish the relations of the articular surfaces. Their fibrous consistence, and the arrest of their development, limit the movements of the replaced joint, and become causes of its re-displacement. The subcutaneous section of these muscles is as indispensibly necessary, as it is in the treatment of club-foot, &c. 2. Muscles which have been passively and consecutively retracted can in certain cases be sufficiently extended by mechanical means. But if this shortening is considerable, and the traction employed too forcible and too long continued, they become fibrous in texture, and serious obstacles to the reduction, and in like manner with the others require division.

3. The fatty transformation, and hypertrophied states of the muscles do not exert any influence upon the reductibility of the dislocations. They may interfere with the movements of the joints after the reduction, but, by the re-establishment of the various relations of the parts, these muscles eventually re-acquire their normal texture and movements.

B. *The Arteries* become flexuous, so as to accommodate themselves to the deviations of the joints from their normal states: they also become diminished in calibre from $\frac{1}{3}$ to $\frac{1}{2}$. It will thus be seen why mischief will not accrue to the arteries by attempts at reduction, and why the limb, even when the reduction has been accomplished, does not at once receive its proportionate development.

C. *The Veins* do not become so tortuous as do the arteries, while their calibre becomes increased in the same proportion as that of the arteries becomes diminished.

D. *The Nerves* are shortened, and stretched in a straighter line than natural between the two extremities of their course, thus opposing some obstacle to, and causing excessive pain during the reduction.

E. *The Cellular Tissue* is generally increased in quantity, and much loaded with fat, which occupies the hollow spaces left under the skin by the displacement of the parts, and the intermuscular spaces. It displaces too the fleshy fibres of the relaxed muscles, so that these almost entirely disappear, or a few of a yellow colour are found amidst a mass of adipose substance.

F. *The Skin* easily accommodates itself by its elasticity, to the changed forms of the parts, and, where this is not the case, fatty matter fills up the intervals.

G. *The Ligaments and Capsules.*—Like the muscles, these are altered in dimension, direction, and structure. They may be contracted or elongated, and in some dislocations their shortening arises from active retraction. In the dislocation of the astragalus outwards, and in the extreme adduction of the foot, the internal lateral ligament of the tibio-tarsal articulation, and the astragalo-scaphoidean ligament, are often found contracted to one-third of their length, hard, and stretched like cords. The ligaments also become shortened by passive retraction after the displacement, and to such an extent as to prevent the reduction by their mere extension. Those ligaments, whose points of attachment have been distanced by the displacement, become lengthened, and thinned, adapting themselves to the parts to which they are attached. Under relaxation and disease they become fatty and wasted, less rapidly however, and in a less degree than do the muscles; and where muscles by reason of excessive traction become fibrous, these parts become osseous. Mere immobility without stretching will sometimes produce ossification. In reference to the reductibility of the dislocations we may observe—1. That ligaments, like muscles, may, by reason of primary or consecutive retraction, oppose invincible obstacles to the reduction, and by reason of their altered state may contribute to the reproduction of the displacement, and therefore require the subcutaneous incision. Moreover, the capsules, especially of the hip-joint, have a concentric retraction which opposes reduction.—2. The elongation of the ligaments and capsules, subsequent to their distention, opposes an obstacle to the permanence of the reduction.

It must be borne in mind that the changes in the soft parts, to which allusion has been made, are developed very gradually, and this remark becomes of importance in reference to a change which especially affects the capsule of the hip-joint. At the examination after death of some old subjects the communication within the capsule between the luxated head and the cavity has been found to be obliterated, and it has been hence inferred that reduction in these cases is impracticable. But observation teaches us, that early in life this state of things does not exist, and, even when it has commenced, it proceeds by very slow steps;—so that it is only towards 12 or 14, that any serious obstacle may be apprehended upon this ground. The communication has been even found open in subjects of 25, 28, and 30 years of age.

B.—*Changes in the Articular Extremities.*

A. *Changes in the Heads of the Bones.*—Taking the head of a femur as an example, its surface often becomes very irregular, its volume is diminished, as also its sphericity, the neck is shortened: its cartilage after undergoing change of structure disappears. A luxated head, by pressure against a part, may become flattened, or, by pressure against the border of the cavity, receive from it an impression or even a groove. The head of the femur is less obliquely placed upon the neck than naturally, while the neck itself is inserted too perpendicularly into the axis of the bone.

B. *Changes in the Cavities.*—These are especially observed in the cotyloid cavities. They have a tendency to diminish in size, and to take on somewhat of the form they possessed in the early stages of intra-uterine development, *i. e.* to become triangular and superficial. An examination of about forty cases, has proved to the author that the diminution of the size of the heads, and the narrowing of the articular cavities are proportionate. So much are these changes the result of the absence of juxta-position, that when an artificial cavity is formed for the reception of a luxated head, the latter continues in its normal proportions, or even may augment these. The cavities have a tendency to become obliterated in proportion to their size and the duration of the deformity. The obliteration seems to arise from two distinct sources, viz. 1. A rising up of the bottom of the cavity, which seems to swell out from the absence of the pressure of the articular head; and—2. From the production of a cellular and fatty structure, which appears to be a degeneration of the normal tissue that occupies the bottom and interstices of all articular cavities. In subluxations, the head of the luxated bone, by pressure upon some one point of the edge of the articular cavity, sinks this down to the level of the surrounding bone, or the cavity becomes enlarged in the direction of the pressure.

In reference to the reductibility we may observe of the above-named changes—

1. When no new articular cavity has been formed, the head diminishes in size, and is changed in form, in much the same proportion as the cavity whence it has escaped, and hence it is possible to bring these parts into mutual relation. But these changes are obstacles to the permanence of the reduction, and to the completeness of the subsequent movements. Yet, the mere restoration of the parts to their natural places, tends greatly to the gradual resumption of their original forms and dimensions.

2. When, by reason of the formation of a new joint, the head has retained its size, or has even increased it, while the old cavity has become narrowed, the want of relation between the parts becomes a source of irreducibility.

3. The various depressions or changes in the heads of the bones, which do not prevent the reduction, yet, may interfere with the solidity of the coaptation, and may impede the movements of the part.

4. The consecutive deformities of the articulations, and the laxity of the capsules, are favorable to the reproduction of the luxation.

All the changes in the articulations themselves, as in those of the soft parts surrounding them, are gradual and slow in their progress, giving rise to two consequences of importance.

1. Although these luxations may become after a given epoch irreducible, yet, they are not so for a length of time before the alterations in these parts have reached their height. The author and other practitioners have reduced a congenital dislocation of ten years' standing, and M. Guillard reduced a dislocation of the humerus in a girl aged 16.

2. The error of the old theory of the production of these dislocations by an arrest or absence of development. It is true, that if we examine an old dislocation of the hip, the head of the femur is mis-shapen and lessened in size, and the cotyloid cavity has become triangular and more or less obliterated; but, if we examine these parts at an earlier period, we find both the head and the cavity unaltered, and that the subsequent changes are only manifested by slow degrees.

C.—*Changes in various Parts in the Vicinity of the Luxations.*

A. *New Articular Cavities.*—Among the subjects of congenital dislocation, there are some in whom new articular cavities are formed, while there are others which reach a very advanced age without this ever taking place. From the examination of numerous preparations, M. Guerin deduces as a law that such new cavities are never formed but after the destruction of the capsules, permitting the head to remain in immediate contact with the bone upon which it lies. As a general observation, such a cavity is never formed prior to the age of 12 or 14, but still there is a great variety in the period at which the capsule ruptures. In an old woman (aged 73), who had a double congenital dislocation of the hip, the capsule on one side was found completely perforated, and the head of the bone secured within a new joint, while, on the opposite side, in which the capsule continued entire, no trace of any new cavity existed, there being simply a depression upon that portion of the ilium upon which the bone rested. After the formation of this new joint, an insurmountable obstacle to the reduction exists, for the ruptured capsule contracts adhesions around the newly-formed surface, and these so firm, as only to be overcome by unjustifiable violence. It also unites with the muscles and surrounding soft parts. The head of the bone too, even prior to the rupture of the capsule, becomes united, by means of a fibro-cellular substance, with the parts with which it lies in contact, forming bridles only to be relieved by the subcutaneous section.

B. *Changes in the Skeleton near the Luxation.*—Changes in form, direction, and dimensions are found in the neighbouring parts of most congenital dislocations, but, it is in that of the hip that these are especially prominent. M. Sedillot was the first to prove the error of the opinion held by Dupuytren, that the *pelvis* did not suffer in these cases. It often does so to a great extent, by reason of the consecutive retraction of the displaced muscles, and the effect of the erect posture. Not only the direction of the various parts of the pelvis becomes changed, but the side upon which the luxation occurs is seized with an arrest of development, and the various foramina and apertures are of consequence smaller upon that side than upon the opposite one. The changes in the pelvis may be regarded under two points of view.

1. As influencing the fixity and solidity of the normal articulations. To a certain extent the consecutive deformities of the pelvis change the direction of the planes of the articular surfaces, and the directions in which they are acted upon by the muscles; and, owing to the admirable natural adaptation of these surfaces, any deviation, however slight, from the normal relations, diminishes the solidity of the parts.

2. They may constitute a consecutive deformity, which will continue, even though the dislocation be reduced; thus, the deformity of the half of the pelvis corresponding to the luxated femur, may be attended with lameness or a difficulty of walking, which may cause even the fact of the reduction of the dislocation to be doubted. The elevation of the pelvis upon the affected side is not permanent, for, if the psoas muscle be brought again into its natural sphere of operation, this will disappear.

In conclusion, we may then state that, under certain conditions, congenital dislocations are reducible; that the probability of their reduction decreases in proportion to the extent of the deformity, and its duration; that they are irreducible when very old, and especially when a new joint has been formed;—and, that the permanence of such reduction is amenable to similar conditions.

### Chap. 2.—*Indications for effecting and consolidating the Reduction of Congenital Dislocations.*

The etiology of the affection at once points out the practical proceedings, and the general therapeutical indications are as follow:—

1. Preparatory and continuous extension of the shortened muscles.
2. The subcutaneous section of those muscles which are unaffected by mechanical extension.
3. The extension or section of the contracted ligaments and capsules.
4. The reduction of the bones to their natural situations.
5. The consecutive treatment, calculated to consolidate the reduction; viz. (1), a mechanical apparatus, destined to maintain the elongation of the muscles which have not been divided, and the separation of the portions of those which have, and to secure the articular surfaces in due relation: (2), the gradual employment of motions calculated to secure the better coaptation of the surfaces, and to re-establish the normal movements of the joint.

Irreducible cases have usually been abandoned as hopeless, but the author recommends, as far as possible, the imitation of the normal conditions of an articulation. This is to be done, by fixing the dislocated extremity upon the nearest possible point to its proper situation, and forming there a new articular cavity, in which the head of the bone may be able to exercise in a limited manner movements somewhat resembling those that are natural to it. In accomplishing this, we must imitate the proceedings of nature, in procuring the perforation of the capsule, the application of the head to its new site, and the adhesion of the ruptured capsule to the border of the projected cavity. Scarifications of the capsule and of that portion of the bone whereon it is desirable to place the head, permit immediate contact, and facilitate future adhesion. The head thus placed, other scarifications must be made around the circumferences of the spot upon which it reposes, and these should be deep in order to encourage the generation of firm fibro-cellular bands: when these last are supposed to be sufficiently strong to offer some resistance, the development of the new cavity, thus marked out, must be sought to be obtained by the means employed by nature, under analogous circumstances; viz. frqeuent but circumscribed movements.

A future work will detail the author's various anatomical and physiological considerations involved in this therapeutical suggestion: he has already acted upon it, with success, in two cases of congenital dislocation of the hip, and one of the clavicle, to the latter of which we have already alluded in this article.*

### IV.—THE INFLUENCE OF THE ATMOSPHERIC PRESSURE UPON THE PRODUCTION OF THE SEROUS EXHALATIONS.

Our limits render it necessary that we should confine ourselves to a brief notice of this very ingenious essay.

The researches of the Webers of Munich† have amply demonstrated, that the head of the femur, during extension, is in a state of accurate approximation with the walls of the cavity, which contains it, and that it is maintained in situ by atmospheric pressure. When the walls of the ace-

* Page 113. † See Baly's Müller's Physiology, p. 962.

tabulum were perforated in their experiments, and the air thus admitted, the extremity at once descended by its own weight. But M. Guerin has observed, in his own experiments, that, during certain movements of the extremity, the intimate adaptation of the surfaces to which we have alluded, does not exist, and, thus, under the influence of these movements, overcoming the effects of the atmospheric pressure, there will be from time to time spaces formed producing a vacuum.

Having observed this fact, he extended his observations to other joints, and he found that in all of them, capable of containing synovia, during the state of extension and repose, a perfect adaptation of the articular surfaces occurred, and, that these were held together by the influence of the pressure of the atmosphere, independently of the muscles and ligaments surrounding them; but also in all of them, during certain motions, the spaces to which allusion has been made were produced. He next considered whether the same circumstances did not prevail with regard to the various serous cavities, and even a slight consideration must prove that such is the case. In all of these, between the parietal layer and the visceral layer, a space exists capable of increase or diminution. That the heart does not fill the *pericardium*, but requires a certain space for its movements, is well known; and, when we consider the attachments of the pericardium, we shall perceive, that during the elevation of the ribs in respiration, its cavity must become enlarged, as it must also during the contraction of the cavities of the heart. The close attachment of the costal and diaphragmatic *pleuræ* to the ribs and diaphragm, and the entire covering they furnish to the lung, are well known, and the extensive space that is left between the parietal and visceral portions is susceptible of change according to the variations induced by the elevation of the thorax, the distention of the lungs, as produced in respiration, speaking, the movements, &c. The defect of correspondence in form and size between the parietal and visceral portions of the *peritoneum* must in like manner give rise to the incessant production or increase of spaces in its cavity. So too, in the *arachnoid*, when the brain is expanded or elevated, the internal cavities become dilated while the portions of the peripheral arachnoid approach each other; when, however, the brain contracts or falls inwards, there is a narrowing of the internal, and an amplification of the peripheral cavities.

The formation of such spaces, or their increase when already existing, during certain movements of the joints and serous cavities, M. Guerin has proved by actual experiments, performed by connecting a tube, partially filled with a coloured fluid, with the interior of these parts, and carefully observing the manner in which the elevation or depression of the fluid, corresponded with the various movements induced.

*Physiological and Pathological Applications.*

These cavities, then, in certain conditions, may be likened to a cupping-glass, in which the air has become rarified and the equilibrium of the atmospheric pressure disturbed. They are thus periodically submitted to a *suction action, which promotes the issue from the vessels of the exhaled fluids.*

There are many observations which confirm this explanation, and are yet themselves explained by it. It has long been known that a greater difficulty attends the motions of the joints, after ascending great elevations, which arises from the diminution of the pressure of the atmosphere. So, too, the difficulty of moving the joints after long immobility is well known, and indeed mere immobility without active disease may lead to anchylosis. By maintaining the layers of the peritoneum in a state of perfect contact, and thus preventing the occurrence of secretion impeding the union, adhesion between these may be produced, which is not the case in regard to mucous membranes. The same ideas explain the ease with which adhesions between the parietes of serous cavities are formed after effusions. The danger attending wounds of the joints and serous cavities has been long known, but its explanation or prevention have not been suggested heretofore ; but now that we are aware of the influence of the pressure of the atmosphere upon the mechanism of the exhalations, the determination of the results which may follow the cessation of this condition is easy. The air, penetrating freely into the serous cavities, then, impedes the mechanism of the secretion of their fluids, which should be induced by a periodical suction, exerted at the orifices of the exhalent vessels ; these vessels, by the stoppage of such exhalation, become overcharged with the stagnated fluids, and the ill-consequences, so often seen, result.

A necessary consequence of this theory, is the institution of rules, which have indeed long prevailed empirically, viz. to endeavour as far as possible to exclude the air from the serous cavities and joints, and to promote its expulsion, as soon as possible, if already admitted.

V. The case of dislocation of the cervical vertebra we noticed some time since,* and a re-perusal of the report does not induce us to change the opinion that we expressed, of its being very doubtful whether any dislocation had really taken place.

M. Guerin's works may be perused with advantage, and although we think that some of his conclusions are too general, and rather too hastily arrived at, yet most of them are well illustrated by experiments, preparations, and allusions to cases which have fallen under his own observation. His theory of the production of deformities gives an intelligible explanation of a large class, the nature of which was heretofore involved in much obscurity ; and his doctrines of subcutaneous surgery, susceptible as they are of an extensive application, demand the earnest attention of operating surgeons.

---

* Medico-Chir. Rev. No. 67, p. 239.

